# Epidémiologie du cancer gastrique: expérience d'un centre hospitalier marocain

**DOI:** 10.11604/pamj.2014.17.42.3342

**Published:** 2014-01-22

**Authors:** Ihsane Mellouki, Nawal laazar, Bahija Benyachou, Nouredine Aqodad, Adil Ibrahimi

**Affiliations:** 1 Service d'hépato gastroentérologie C4, CHU Hassan II, Faculté de médecine et de pharmacie, université Sidi Mohammed Ben Abdallah, Route Sidi hrazem, CP 30000, Fès Maroc

**Keywords:** Cancer de l'estomac, épidémiologie, pronostic, Helicobacter pylori, Stomach cancer, epidemiology, prognosis, Helicobacter pylori

## Abstract

Le cancer de l'estomac est représenté essentiellement par Les adénocarcinomes gastriques, ces derniers demeurent l'une des dix premières causes mondiales de mortalité avec un pronostic qui est péjoratif. Son incidence reste variable à travers le monde, elle est caractérisée par une importante disparité géographique. Le but de notre travail est de décrire les caractéristiques épidémiologiques de l'adénocarcinome gastrique dans notre contexte à travers une étude rétrospective, observationnelle étalée sur une période de 10 ans (Janvier 2001- Janvier 2011), incluant tous les malades admis au service d'hépato-gastroentérologie du CHU Hassan II de Fès pour prise en charge d'un adénocarcinome gastrique. Durant cette période, 343 patients étaient admis pour prise en charge d'une tumeur gastrique, dont 170 patients avaient un adénocarcinome gastrique (49.5%). L’âge moyen de ces patients était de 58±13.4 ans [16 ans-0 ans]. Dans 43.7% des cas, les patients provenaient de la région de Fès, souvent du milieu rurale. On note une nette prédominance masculine, avec une différence significative entre les 2 sexes (p < ;0.05). Les patients âgés de moins de 60ans représentaient la tranche d’âge prédominante (63%) par rapports aux patients âgés de plus de 60ans (p = 0.02). 61% des patients consultaient dans un délai allant de 1 mois à 6 mois, 30.4% des patients étaient tabagiques, ce facteur avait une relation statistiquement significative avec l'adénocarcinome gastrique (p = 0.02). la non consommation de l'alcool est inversement liée et de façon significative à l'apparition de l'adénocarcinome gastrique (p = 0.03) dans notre contexte. L'infection par Hélicobacter pylori n’était mentionnée que chez peu de malades. Les formes métastatiques au moment du diagnostic dépassaient 50% avec un taux de décès au cours de l'hospitalisation de 2.6%. Sur le plan endoscopique, la localisation antropylorique, et la forme ulcéro-végétante étaient prédominantes, elles présentaient successivement 49% (p = 0,002) et 66% (p = 0.00001). Une chirurgie curative n’était proposée que chez 50 patients (30.2%). L'adénocarcinome gastrique représente le type histologique le plus fréquent, son pronostic reste fâcheux dans notre région, touchant une population jeune, minimisant ainsi les chances de tout traitement curatif.

## Introduction

L′adénocarcinome gastrique se développe à partir de l′épithélium gastrique. Son incidence est en diminution depuis 50 ans dans les pays occidentaux. Cependant, bien que leur incidence annuelle ait notablement décrue, il demeure l'une des dix premières causes mondiales de mortalité liée au cancer [1], il représente la deuxième cause de mortalité par cancer dans le monde [2–4]. Son incidence est caractérisée par une importante disparité géographique, ainsi, l'Afrique est une région à faible risque du cancer gastrique, l'Europe occidentale et l'Amérique du Nord sont des régions à risque moyen, et l'incidence la plus élevée est rapportée au Japon, suivi de la Chine, l'Amérique du Sud et l'Europe de l'Est et du Sud [1]. Plusieurs études épidémiologiques ont été menées afin d'identifier les différents facteurs de risque du cancer gastrique, montrant que c'est un cancer multifactoriel, et démontrant le rôle important de l'infection par Hélicobacter pylori [6].

## Méthodes

Nous avons réalisé une étude rétrospective observationnelle étalée sur une période de 10ans (Janvier 2001-Janvier 2011) de l'ensemble des patients admis au CHU Hassan II de Fès pour prise en charge d'un cancer gastrique. L'inclusion avait concerné 170 patients chez qui le diagnostic d'un adénocarcinome gastrique a été retenu. C'est une étude descriptive des différents caractères épidémiologiques (âge, sexe, antécédents), des facteurs de risque de l'adénocarcinome gastrique, des différentes caractéristiques de la tumeur (localisation, aspect macroscopique, métastases), ainsi que l’évolution des patients.

Les variables quantitatives ont été décrites en termes de moyenne et d’écart type et les variables qualitatives en termes de pourcentage. Pour comparer deux groupes, les tests paramétriques classiques (Test de Khi2, test de Student, ANOVA) ont été utilisés. Pour chaque test statistique utilisé, le test a été considéré comme significatif lorsque p (degré de signification) était inférieur à 0.05. L'analyse statistique a été effectuée en utilisant les logiciels Epi-Info version 2003 et le logiciel SPSS (version 17).

## Résultats

Durant cette période d’étude, 343 patients étaient admis au service d'hépato-gastroentérologie du CHU Hassan II de Fès pour prise en charge d'une tumeur gastrique, dont 170 patients avaient un adénocarcinome gastrique (49.5%). L’âge moyen de ces patients était de 58 + /-13.4 ans [16 ans-90 ans]. Dans 43.7% des cas, les patients provenaient de la région de Fès, souvent du milieu rurale. On note une nette prédominance masculine, 48 femmes (28%) et 122 hommes (72%), avec un sex-ratio de 2.5 et une différence significative entre les 2 sexes (p < 0.05). Les patients âgés de plus de 60ans représentaient la tranche d’âge prédominante dans 63% par rapports aux patients âgés de moins de 60ans (p = 0.02). 61% des patients consultaient dans un délai allant de 1 mois à 6 mois. Concernant les facteurs de risque de l'adénocarcinome gastrique, le tabagisme actif était retrouvé chez 30.4% des patients, ce facteur avait une relation statistiquement significative avec l'adénocarcinome gastrique (p = 0.02). La non consommation de l'alcool est inversement liée et de façon significative à l'apparition de l'adénocarcinome gastrique (p = 0.03). L'infection par Hélicobacter pylori n’était mentionnée que chez peu de malades. Les formes métastatiques au moment du diagnostic dépassaient 50% avec un taux de décès au cours de l'hospitalisation de 2.6%.

Le mode de révélation de l'adénocarcinome gastrique dans notre population prenait différents aspects cliniques, ainsi la douleur épigastrique présentait le syndrome prédominant chez 75% des patients, une hémorragie digestive était le symptôme révélateur chez 33 malades (19%), une dysphagie chez 19 patients (11%) révélant la localisation cardiale chez 9 patients, une altération de l’état générale avec asthénie, anorexie et amaigrissement était présente chez 54 malades.

Sur le plan endoscopique, la localisation antropylorique prédominait par rapport aux autres localisations dans 49% (p = 0.002). L'aspect macroscopique en endoscopie était dominé par la forme ulcéro-végétante dans 66% (p = 0.00001). Une chirurgie curative n’était proposée que chez 50 patients (30.2%).

## Discussion

Le cancer gastrique constitue le 2^ème^ cancer chez l'homme et le 3^ème^ cancer chez la femme en Asie et à travers le monde [7, 8]. En se basant sur les données du GLOBOCAN 2008, le cancer gastrique est le second cancer digestif après les cancers colorectaux et la quatrième cause de décès par cancer [8]. Une méta-analyse des cancers gastriques en Afrique a montré une franche augmentation de l'incidence de ce cancer au Mali (20,3/100000) par rapport au autres pays d'Afrique, ainsi qu'une incidence plus augmentée en Afrique Sub saharienne par rapport à l'Afrique du nord [9].

Le cancer gastrique touche plus les hommes que les femmes aussi bien en Afrique que dans les autres continents. Une étude coréenne n'avait pas retrouvé de différence statistiquement significative entre les 2 sexes [10], contrairement à ce qui ressort dans notre étude où nous avons noté une prédominance masculine significative (p < ;0.05). Le cancer gastrique survient rarement avant l’âge de 40 ans, l'incidence augmente rapidement au-delà avec un pic pendant la septième décennie [1]. Résultat qui ressort également dans la même étude coréenne où l'incidence augmente avec l’âge avec une augmentation particulière à partir de 60 ans [10], ainsi que dans notre étude où les patients âgés de plus de 60 ans représentaient la tranche d’âge prédominante dans 63% par rapports aux patients âgés de moins de 60 ans (p = 0.02).

Le mode de déclaration des adénocarcinomes gastriques est polymorphe, pouvant aller de la simple gêne épigastrique, simulant parfois la douleur de type ulcéreuse ou le syndrome de masse tumorale épigastrique dans les formes avancées, ce qui ressort également dans notre série où plus de 50% des patients consultaient à un stade métastatique. La survie à 5 ans est inférieure à 30% dans les pays développés, et inférieure à 20% dans les pays en voie de développement [7, 11].

L'adénocarcinome gastrique est un cancer dont l'incidence est variable à travers le monde. Les incidences les plus élevées ont été décrites en Asie de l'Est (Japon, chine et Korée). En effet, au Japon, une incidence de 102040 nouveau cas a été rapportée en 2008[5]. De même, une étude japonaise réalisée par le ministère de la santé avait montré que 50000 hommes et femme décèdent chaque année par un cancer gastrique, ce qui représente approximativement 15% de mortalité annuelle liée au cancer au cours des 4 dernières décennies [12]. L'Europe occidentale et l'Amérique du Nord sont des régions à risque moyen. En France, en 1992, l'incidence annuelle du cancer gastrique était de 11.1/100000 chez l'homme, et de 4/100000 chez la femme. L'Afrique représente une région à faible risque de cancer gastrique [1]. L'incidence mondiale a nettement diminué au cours de la 2^ème^ moitié du 20^ème^ siècle, elle concerne le cancer de l'estomac distal, de l'antre et du corps, cependant, celle du cancer du cardia reste controversée, plusieurs études suggèrent une augmentation de son incidence [1, 13, 14, 15]

Plusieurs facteurs environnementaux, génétiques et un certain nombre d'affections ont été incriminés comme facteurs étiopathogéniques dans la survenue du cancer gastrique. Parmi les facteurs environnementaux, les facteurs alimentaires jouent un rôle important dans la cancérogenèse gastrique. Ainsi la consommation importante de sel et l'exposition aux nitrosamines est associé à un risque accru de cancer gastrique, ce qui explique l'incidence élevée en Asie du Sud [1, 5, 10], la consommation quotidienne de sel de la population coréenne était de 13.4g en 2005 [9, 16], alors que la dose recommandée par l'organisation mondiale de la santé est de 5g [17]. Le tabagisme a été prouvé également comme facteur de risque de survenue de cancer gastrique, il était classé par l'agence internationale de recherche des cancer (IARC) comme carcinogène du groupe 1 au niveau gastrique, plusieurs études coréennes avait montré une association entre la durée du tabagisme et l'incidence et la mortalité liée au cancer gastrique [19, 18, 20], ce risque est surtout accru en cas d'association avec l'infection par Hélicobacter pylori (HP)[1]. La consommation tabagique était présente de façon significative dans notre série (p = 0.02), dans une étude allemande, aucune relation significative n'a été trouvé entre le tabac et le cancer gastrique [21].

Concernant l'alcool, les études n'ont pas montré un rôle clairement établi de ce facteur dans la survenue du cancer gastrique [1]. Hélicobacter pylori a été prouvé par différentes études mondiales comme facteur de risque du cancer gastrique, il est classé par l'IARC comme facteur carcinogène, avec un risque relatif de cancer gastrique de 2 à 6 fois plus élevé chez les patients infectés par l'HP par rapport à une population non infectée [1, 7], la prévalence la plus élevée était rapportée en Inde où elle varie de 56% à 89% [7]. En Afrique ou la prévalence de l'HP est augmentée, variant entre 70% et 92%. Par contre l'incidence du cancer gastrique reste néanmoins intermédiaire. Ce qui impose à ce jour la réalisation de travaux permettant de rechercher de façon précise cette association dans notre contexte [9].

Des facteurs génétiques peuvent aussi être incriminés dans la survenue du cancer gastrique, ces facteurs sont suggérés vue d'une part l'existence d'un risque multiplié par 2 ou 3 chez les apparentés du premier degré d'un sujet atteint, et d'autre part, vue le polymorphisme d'une grande variété de gènes susceptibles de modifier l'effet de l'exposition aux carcinogènes environnementaux, cette susceptibilité peut être impliquée à différentes étapes de carcinogenèse (protection de la muqueuse gastrique, réponse inflammatoire, détoxification des carcinogènes, oncogène)[1]. Le cancer gastrique est de mauvais pronostic, dans notre série, 50% des patients étaient admis à un stade métastatique, et la chirurgie curative n’était proposée que chez 30%. Dans une étude réalisée à partir du registre des cancers digestifs de la côte d'or sur une période de 19 ans, la survie à 5ans était de 14.4%, et seul 14.4% des patients avaient bénéficié d'une chirurgie curative [22].

## Conclusion

L'adénocarcinome gastrique représente le type histologique le plus fréquent, son pronostic reste fâcheux dans notre région, touchant une population jeune, minimisant ainsi les chances de tout traitement curatif. Ces constations nous mènent en faite à poser plusieurs questions sur l'incidence du cancer gastrique dans notre pays caractérisé par une grande diversité des habitudes alimentaires, et où la prévalence de Hélicobacter pylori dépasse les 70% chez les patients ulcéreux. L'intérêt du dépistage de l'adénocarcinome gastrique doit être évalué, une étude japonaise s'intéressant au dépistage annuel du cancer gastrique chez les patients de plus de 50 ans par endoscopie, a permis de diagnostiquer 40% des tumeurs à un stade superficiel et de diminuer la mortalité spécifique du au cancer gastrique [1].
